# Relation between smoking history and gene expression profiles in lung adenocarcinomas

**DOI:** 10.1186/1755-8794-5-22

**Published:** 2012-06-07

**Authors:** Johan Staaf, Göran Jönsson, Mats Jönsson, Anna Karlsson, Sofi Isaksson, Annette Salomonsson, Helen M Pettersson, Maria Soller, Sven-Börje Ewers, Leif Johansson, Per Jönsson, Maria Planck

**Affiliations:** 1 Department of Oncology, Clinical Sciences, Lund University and Skåne University Hospital, Barngatan 2:1, SE-22185, Lund, Sweden; 2 CREATE Health Strategic Center for Translational Cancer Research, Lund University, BMC C13, SE 221 84, Lund, Sweden; 3 Center for Molecular Pathology, Department of Laboratory Medicine, Lund University, SE 20502, Malmö, Sweden; 4 Department of Clinical Genetics, Lund University and Regional Laboratories Region Skåne, SE 22185, Lund, Sweden; 5 Department of Pathology, Lund University and Regional Laboratories Region Skåne, SE 22185, Lund, Sweden; 6 Department of Thoracic Surgery, Clinical Sciences, Lund University and Skåne University Hospital, SE 22185, Lund, Sweden

**Keywords:** Lung cancer, Smoking, Gene expression analysis, Adenocarcinoma, EGFR, Never-smokers, Immune response

## Abstract

**Background:**

Lung cancer is the worldwide leading cause of death from cancer. Tobacco usage is the major pathogenic factor, but all lung cancers are not attributable to smoking. Specifically, lung cancer in never-smokers has been suggested to represent a distinct disease entity compared to lung cancer arising in smokers due to differences in etiology, natural history and response to specific treatment regimes. However, the genetic aberrations that differ between smokers and never-smokers’ lung carcinomas remain to a large extent unclear.

**Methods:**

Unsupervised gene expression analysis of 39 primary lung adenocarcinomas was performed using Illumina HT-12 microarrays. Results from unsupervised analysis were validated in six external adenocarcinoma data sets (n=687), and six data sets comprising normal airway epithelial or normal lung tissue specimens (n=467). Supervised gene expression analysis between smokers and never-smokers were performed in seven adenocarcinoma data sets, and results validated in the six normal data sets.

**Results:**

Initial unsupervised analysis of 39 adenocarcinomas identified two subgroups of which one harbored all never-smokers. A generated gene expression signature could subsequently identify never-smokers with 79-100% sensitivity in external adenocarcinoma data sets and with 76-88% sensitivity in the normal materials. A notable fraction of current/former smokers were grouped with never-smokers. Intriguingly, supervised analysis of never-smokers versus smokers in seven adenocarcinoma data sets generated similar results. Overlap in classification between the two approaches was high, indicating that both approaches identify a common set of samples from current/former smokers as potential never-smokers. The gene signature from unsupervised analysis included several genes implicated in lung tumorigenesis, immune-response associated pathways, genes previously associated with smoking, as well as marker genes for alveolar type II pneumocytes, while the best classifier from supervised analysis comprised genes strongly associated with proliferation, but also genes previously associated with smoking.

**Conclusions:**

Based on gene expression profiling, we demonstrate that never-smokers can be identified with high sensitivity in both tumor material and normal airway epithelial specimens. Our results indicate that tumors arising in never-smokers, together with a subset of tumors from smokers, represent a distinct entity of lung adenocarcinomas. Taken together, these analyses provide further insight into the transcriptional patterns occurring in lung adenocarcinoma stratified by smoking history.

## Background

Due to high incidence and poor survival, lung cancer is the worldwide leading cause of death from cancer. Small cell lung cancer accounts for about 15% of all lung cancer diagnoses whereas non-small cell lung cancer constitutes the majority of cases, primarily including adenocarcinoma (AC) and squamous cell carcinoma. Although the use of cigarettes is the major pathogenic factor, not all cases of lung cancer can be attributable to smoking [1]. Lung cancer in never-smokers has been suggested to represent a different disease entity compared to lung cancer arising in smokers [2,3]. Specifically, lung cancer in never-smokers has been associated with female sex, East Asian ethnicity, AC histology, differences in mutational pattern of *EGFRKRAS,* and *TP53*, and response to EGFR inhibitors [2-4]. However, despite numerous reports of gene expression derived AC subtypes [5-10], a distinct subtype comprising only or predominantly of never-smokers has not been identified. Taken together, this warrants further investigation of the transcriptional differences between AC arising in never-smokers and smokers.

In the present study, we aimed to delineate transcriptional differences between never-smokers and current/former smokers with AC by both unsupervised and supervised gene expression analysis, combined with conventional molecular assays, measurements of pathway activation by different gene expression metagenes, and histopathological data, across several AC data sets.

## Methods

### Ethics statement

The study was approved by the Regional Ethical Review Board in Lund, Sweden (Registration no. 2004/762 and 2008/702). Written informed consent was obtained from all patients diagnosed after 2004, whereas for the retrospective part of the material, i.e. patients diagnosed earlier than 2004, study inclusion was approved by the Regional Ethical Review Board in Lund, Sweden, if patients (or their family members/survivors) not stated otherwise when they were informed about the study in 2006.

### Patient material

39 AC were obtained from patients selected for surgery of early stage, primary lung cancer between 1989–2007 at the Skåne University Hospital, Sweden. Smoking history was obtained from patient charts and included 13 current smokers, 14 former smokers, and 10 never-smokers. Among the former smokers four patients quit smoking less than one year before surgery. None of the patients had received neoadjuvant treatment prior to surgery. Within an hour after lobectomy/pulmectomy, a biopsy from a macroscopically representative area of the tumor was selected by a lung cancer pathologist (most often LJ) and freshly frozen in −80 °C. DNA and RNA were subsequently extracted from the freshly frozen specimens, according to published protocols [11]. Tumor histology of all original tumor blocks was confirmed by a lung cancer pathologist (LJ). With the exception of one node positive (N1) and one with non-evaluable N-status, all cases were T1-4N0M0. Clinical and histopathological data are summarized in Table 1.

**Table 1:** Baseline data for used AC cohorts

	**Illumina**	**DCC**	**GSE10072**	**GSE12667**	**Beer et al.**	**GSE32863**	**GSE11969**
Data set type	Original cohort	External	External	External	External	External	External
Microarray platform	Illumina HT12	Affymetrix U133A	Affymetrix U133A	Affymetrix U133 2plus	Affymetrix HU6800	Illumina WG6	Agilent
Number of AC cases*	39***	349	58	51	81	58	90
Stage IA/IB/IIA/IIB/IIIA	10/18/4/3/2	84/133/16/64/45	5/17/3/18/9	2/9/0/2/2	NA	16/18/9/2/12	28/24/4/9/20
Median age (years)	69 (36–83)	65 (35–87)	67.5 (45–81)	NA	64 (41–85)	70 (39–86)	62 (32–84)
Female/Male	21/18	187/162	23/35	24/27	47/34	45/13	43/47
Never-smokers/Smokers	10/27	49/300	16/42	8/43	9/72	29/29	45/45
Never/Current/Former smokers	10/13/14	49/32/268	16/24/18	8/18/25	9/NA/NA	29/29/0	45/NA/NA
Percentage never-smokers	27%	14%	28%	16%	11%	50%	50%
Never-smokers (Female/Male)	6/4	40/9	13/3	6/2	9/0	23/6	35/10
*EGFR* mutation/wt	4/35	NA	NA	NA	NA	NA	32/58
*KRAS* mutation/wt	12/27	NA	NA	NA	36/44	NA	10/80
P53 +/wt	NA	NA	NA	NA	16/64	NA	29/61
Mean follow-up OS (years)**	6.2 (0–15.5)	4.3 (0–17)	NA	NA	3 (0.1–9.2)	NA	5.5 (0.5–9)

* Number of cases with smoking history.

** OS: Overall survival.

***: Two cases are not annotated for smoking.

### External lung AC expression data sets

The DCC [12] (n = 444, Affymetrix U133A), GSE10072 [13] (n = 58, Affymetrix U133A), GSE12667 [14] (n = 75, Affymetrix U133 2 plus), Beer et al. [7] (n = 86, Affymetrix HU6800), GSE32863 (n = 58, Illumina WG6 version 3), and GSE11969 [9] (n = 158 including 90 AC, Agilent GPL7015) gene expression data sets were used for supervised analysis and to validate the gene signature derived from unsupervised analysis. The GSE7895 [15] (n = 104, Affymetrix U133A), GSE19027 [16] (n = 52, Affymetrix U133A), GSE19667 [17] (n = 121, Affymetrix U133 2 plus), GSE11952 [18] (n = 83, Affymetrix U133 2 plus), GSE32863 (n = 58, Illumina WG6 version 3), and GSE10072 (n = 49, Affymetrix U133A) data sets were used to investigate the gene signature from unsupervised analysis in histologically normal bronchial airway epithelial cells or normal adjacent lung tissue (GSE32863 and GSE10072). Only probe sets present on the U133A chip were used for U133 2 plus arrays in all analyses. Affymetrix data sets were MAS5 normalized and updated for probe annotations as described [19] and individually mean-centered. Normalized expression data for GSE11969 were converted to log2 scale and mean-centered using all 158 samples from GEO. Normalized expression data for GSE32863 was obtained from GEO and were mean-centered using either all AC samples, or all normal samples respectively. Only samples in external data sets with smoking annotations were used in comparisons. Clinical and histopathological data for cases in external data sets are summarized in Table 1. Never-smoking patient history was inferred if a specific annotation existed, and/or if pack-years were equal to zero (Beer et al. and GSE11969). Pack-year data for smokers were available for GSE11969, Beer et al. GSE32863, GSE19027, GSE7895, GSE19667 and GSE11952.

### Unsupervised gene expression analysis

Unsupervised gene expression analysis was performed on a set of 39 AC analyzed by Illumina Human HT-12 V3 microarrays (Illumina, San Diego, Ca). Total RNA was labeled in a 96-well format using the Total Prep-96 RNA amplification kit, hybridized and scanned according to manufacturer’s instructions. Seventy-two lung carcinomas of various histologies were profiled similarly and quantile normalized gene expression data were extracted for all 39 AC cases from this cohort. Gene expression data for the 72 cases is available through Gene Expression Omnibus [20] (GEO) as series GSE29016. Normalized gene expression data for the 39 AC cases were subsequently mean-centered across tumors for each probe. Probes with standard deviation >1 of expression (log2ratio) across samples were used in unsupervised analyses. Hierarchical clustering was performed in MeV [21] using Pearson correlation and complete linkage. Significance Analysis of Microarrays (SAM) analysis [22], performed in MeV, was used to identify genes discriminating between groups identified from unsupervised analysis. A centroid-based gene expression signature was constructed based on discriminating genes from SAM analysis between the two clusters identified by unsupervised analysis of AC cases. Centroid values for each gene correspond to the average expression of the gene across samples in each group. Illumina probes in the gene expression signature were merged on gene identifier prior to validation in external data sets. When multiple Agilent or Affymetrix probe sets from external data sets matched a gene in the gene signature, the probe set with the highest log2ratio standard deviation across samples was selected to represent the gene. Classification of samples was performed by calculating Pearson correlations between samples and centroids, assigning samples to the gene expression centroid with the highest correlation. The latter implies that there are no unclassified samples.

### Supervised gene expression analysis based on smoking history

Supervised analyses between never-smokers and smokers (current or former) were performed for the original Illumina cohort and the DCC, GSE11969, GSE10072, GSE12667, Beer et al., and GSE32863 data sets. For each data set probes/probe sets with log2ratio standard deviation >1 across samples were identified and used in SAM analysis performed in MeV of annotated never-smokers versus smokers. Probes with false discovery rate < 5% from SAM analysis were used to create a never-smoker and a smoker gene expression centroid. Due to the fixed false discovery threshold centroid probe numbers differed between data sets. To ensure that sufficient number of up-regulated/down-regulated probes were present in the centroids for the correlation analyses, centroids were checked for number of up- or down-regulated genes. If a centroid contained < 20 probes with log2 ratio fold change <0, or >0, respectively, then probes with higher false discovery rate were added to the centroids (up to 20 probes in either direction). Centroids for a data set were subsequently used to classify all seven data sets into either smokers or never-smokers. Probes/probe sets in gene expression signatures were merged on gene identifier prior to validation in other data sets. When multiple Agilent, Affymetrix or Illumina probe sets matched a gene in a gene signature, the probe set with the highest log2ratio standard deviation across samples was selected to represent the gene. Classification of samples was performed by calculating Pearson correlations between samples and centroids, assigning samples to the gene expression centroid with the highest correlation. The latter implies that there are no unclassified samples. To investigate the effect of different classification thresholds, we also applied fixed Pearson correlation cut-offs for the DCC-derived centroid classifier, ranging from 0 (all samples classified) to 0.4. This introduced unclassified samples with increasing cut-offs.

### Gene expression metagenes for measuring activation of different pathways

A gene expression metagene for proliferation was created by taking the average log2ratio of genes in the CIN70 signature [23]. Gene expression metagenes for 27 cellular processes originally reported by Bryant et al. [5], referred to as pathways hereon, were computed as described [5]. For external Affymetrix data sets the pathway probe set annotations from Bryant et al. [5] were used to compute mean pathway expression, otherwise matching was made based on gene symbol.

### Functional pathway analysis

Functional analysis was performed using LitVAn [24] and the Ingenuity Pathway Analysis (IPA) software (Ingenuity Systems Inc, Redwood City, CA). For IPA, a p-value < 0.05 for a canonical pathway was considered significant.

### Immunohistochemistry

Immunohistochemical (IHC) staining was performed on 3 μm sections after deparaffinization and rehydration. Heat induced antigen retrieval was performed in low pH buffer (PTEN, Dako S1699), high pH buffer (pAKT, Dako S2367) or TE buffer (CD117/cKIT). Antibodies were obtained from either Cell Signaling Technology (PTEN; 1:100 dilution) or Dako (pAKT; 1:15 dilution, CD117/cKIT; 1:500 dilution). Stainings were visualized using Envision™ (pAKT, CD117/cKIT) or LSAB™ (PTEN) (Dako). EGFR were stained using the mouse monoclonal anti-human EGFR clone 2- antibody and the EGFR pharmDX kit (Dako). After IHC staining, sections were counterstained with hematoxylin, dehydrated and mounted.

### Mutation analysis

*KRAS* mutations were investigated using the TheraScreen K-ras mutation kit (Qiagen). The assay was performed according to the manufacturers’ instructions on a Rotor Gene 3000 instrument (Corbett Research). Mutations of exon 18 through 21 of the *EGFR* gene and of exons 9 and 20 of the *PIK3CA* gene were analyzed by direct DNA sequencing using the BigDye Terminator Cycle Sequencing Kit v1.1 (Applied Biosystems). Sequencing products were separated by capillary electrophoresis in an ABI 3130xl Genetic Analyzer (Applied Biosystems) and the sequence curves were analyzed using the 3100 data collection software (Gene Code Corporation). All sequence alterations were confirmed after a repeated extraction of DNA.

### Quantitative real time-PCR

Quantitative real time-PCR was performed using Rotor Gene 3000 (Corbett Research) and the binding dye iTaqTM SYBR® Green Supermix (BIO-RAD). To determine the copy number of the *EGFR* gene we used the genes for albumine and glucokinase as controls. The ratios were compared to similar ratios of control DNA. A standard curve for each run was constructed from serial dilutions. The CT-threshold was set to 0.2. Amplification mixes (20 μL) contained 10 ng sample DNA, 10μL binding dye, 1μL primer and dH2O. Thermal cycling conditions comprised 10 min at 95 °C and 45 cycles at 95 °C for 15 s, 55 °C at 30s and 72 °C at 30s. All the samples were analyzed in triplicate and the serial dilutions were performed in duplicates. Relative gene copy numbers were calculated using the Pfaffl method representing average values of *EGFR* gene copy numbers in relation to albumin and glucokinase. Ratio ≥1.5 signified amplification.

## Results

### Unsupervised gene expression analysis identifies subgroups of lung adenocarcinoma associated with smoking history

**Figure 1:**
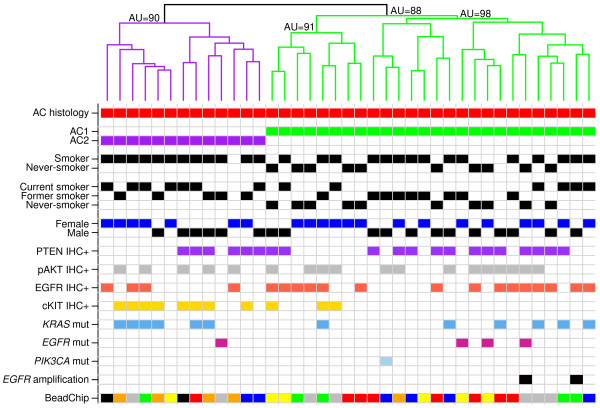
**Molecular profiling identifies two subgroups of lung adenocarcinoma with differences in smoker status.** Unsupervised analysis of 39 AC cases identifies two AC groups, where one (AC1) comprises all never-smoker cases. Hierarchical clustering was performed on 706 Illumina probes with log2ratio SD >1 across the 39 cases using Pearson correlation and complete linkage. The color-coded beadchip annotation bar indicates which of the seven beadchips that a specific sample was hybridized to. Approximately Unbiased (AU) p-values (%) provided by pvclust [35] analysis using 1000 bootstraps are indicated for certain branches of the hierarchical tree, where values close to 100% indicate that clusters are highly supported by data.

To investigate whether AC arising in never-smokers display marked transcriptional differences compared to AC arising in smokers, we first performed unsupervised analysis of 39 well-characterized AC with a comparatively high proportion of never-smokers (Table 1). Intriguingly, this analysis identified two main subgroups, referred to as AC1 and AC2 hereon, of which one (AC1) harbored all never-smokers (n = 10) together with 56% of smoker cases (p = 0.02, Fisher’s exact test) (Figure 1). Moreover, never-smokers within AC1 did not form an apparently distinct subgroup compared to current or former smokers in the same cluster (Figure 1). Since, stratification of smokers into current and former revealed no significant differences between AC1 and AC2, with 46% of all current smokers and 64% of all former smokers being classified as AC1 (p = 0.45, Fisher’s exact test) (Figure 1), we refer to current/former smokers as smokers hereon if not stated otherwise. In addition, of the four former smokers that quit smoking <1 year before surgery, 50% were found in AC1 and 50% in AC2. To further characterize AC1 and AC2 we used available clinical and molecular data for the 39 AC cases. Two main differences between the two subgroups were observed: 1) a strong association of AC2 with positive cKIT IHC staining (p = 0.003, Fisher’s exact test) and *KIT* mRNA overexpression (p < 0.00001, Wilcoxon’s test), and 2) association of AC1 with an increased EGFR activity compared to AC2. The latter was supported by: 1) three of four *EGFR* mutations and the two *EGFR* amplifications were found in AC1 (Figure 1), 2) elevated mRNA expression level of an EGFR metagene [5] in AC1 cases compared to AC2 (p = 0.01, Wilcoxon’s test), and 3) a trend for association of positive EGFR IHC staining with AC1 (p = 0.17, Fisher’s exact test). Notably, expression levels of the *KIT* gene and the EGFR metagene differed between: a) smokers in AC1 compared to AC2 (p = 0.0002 and 0.04, respectively Wilcoxon’s test), b) between AC1 never-smokers and AC2 smokers (EGFR-metagene trend-like) (p < 0.00001 and 0.12, respectively Wilcoxon’s test), but not between smokers vs never-smokers within AC1 (p > 0.05, Wilcoxon’s test). In contrast, the two AC subgroups were not associated with differences in *KRAS* mutation status, PTEN IHC status, pAKT IHC status, gender or overall survival (OS) (p > 0.05, Fisher’s exact test, Wilcoxon’s test, log-rank test). The CIN70 metagene expression, used as a proliferation estimate, did not differ between AC1 and AC2 in general, or between smokers within subgroups (p > 0.05, Wilcoxon’s test). However, within the AC1 group, as well as in a general comparison, never-smokers showed lower expression of the CIN70 metagene compared to smokers (p = 0.01,and p = 0.007 respectively, Wilcoxon’s test).

### Validation of the association of adenocarcinoma subgroups with smoking history in external AC data sets

To validate the association between the AC1 gene expression pattern and never-smoking status we first delineated transcriptional differences between the original AC clusters by SAM analysis. This analysis identified 176 differentially expressed probes, representing 140 genes, at a false discovery rate of <3.5% between the two subgroups. Next, we constructed a centroid-based gene expression signature using significant probes from the SAM analysis for validation in external gene expression studies (in Additional file 1: Table S1). We applied this signature to the DCC, GSE10072, Beer et al., GSE11969, GSE12667, and GSE32863 gene expression data sets comprising a total of 811 AC cases, of which 687 had available smoking status. These 687 cases represent tumors of various stage, differentiation, patient ethnicity, sex, and age, and, notably, have been analyzed by different microarray platforms. Convincingly, the gene expression signature showed high sensitivity (79–100%) in classifying cases annotated as never-smokers as AC1 in all data sets (Table 2). Also, the proportions of smokers classified as AC1 in the AC data sets were similar to our original data (Table 2). Stratification of AC smokers into former or current smokers for the DCC, GSE10072, GSE32863 and GSE12667 data sets showed that 55–67% of former smokers and 31–46% of current smokers were classified as AC1, again representing similar proportions as in our original data set (Table 2). In addition, analysis of pack-year data from Beer et al., GSE32863, and GSE11969 revealed no significant difference between smokers in AC1 compared to AC2 (p > 0.05 all comparisons, Student’s *t*-test).

**Table 2:** Association of AC1/AC2 subgroups in external AC data sets with smoking history

**Data set**	Nbr NS/smokers*	Nbr NS/CS/FS*	**Nbr NS classified as AC1**	**% smokers classified as AC1 (All/CS/FS)**	Fisher’s exact test P-value **
DCC	49/300	49/32/268	45 (92%)	53/31/55	4 × 10^−8^/0.01
GSE10072	16/42	16/24/18	15 (94%)	55/46/67	0.005/0.22
GSE12667	8/43	8/18/25	8 (100%)	49/33/60	0.007/0.12
Beer et al.	9/72	9/NA/NA	8 (89%)	53/NA/NA	0.07/NA
GSE11969	45/45	45/NA/NA	37 (82%)	58/NA/NA	0.02/NA
GSE32863	29/29	29/29/0	23 (79%)	38/38/NA	0.003/NA

* NS: never-smoker, CS: current smoker, FS: former smoker.

** Calculated from a 2×2 contingency table summarizing number of never-smokers and smokers for each AC subgroup (first p-value), or number of CS and FS for each AC subgroup (second p-value).

### Comparison of adenocarcinoma subgroups with results from supervised analysis based on smoking history

Given the high sensitivity but low specificity of the AC1/AC2 classification in identifying never-smokers we sought to further investigate transcriptional differences between never-smokers and smokers. Using supervised gene expression analysis in the original 39 AC cohort and six validation data sets we generated never-smoker and smoker gene expression centroids for each data set (in Additional file 1: Table S1). Centroids for a data set were subsequently used to classify all other data sets into either smokers or never-smokers (see Methods). The centroid classifiers derived from the DCC and GSE10072 data sets showed the best performance in correctly identifying never-smokers across different data sets (Table 3), with performance similar to validation results from the unsupervised analysis (Tables 2 and 3). Similar to the AC1 and AC2 classification a high percentage of smokers were classified as never-smokers by all centroid classifiers across data sets (14–60%) (in Additional file 2: Table S2). Moreover, stratification of smokers into current and former smokers revealed that notable fractions of both current (4–52%) and former smokers (28–61%) were classified as never-smokers by the centroid classifiers across data sets (in Additional file 2: Table S2). Notably, the classifier from GSE32863 generated from comparison of 29 never-smokers and 29 current-smokers did not perform better than classifiers from the DCC and GSE10072 data sets constructed from comparison of never-smokers versus a mix of current and former smokers (Table 3). To further explore this finding we also constructed a classifier from SAM analysis of current versus never-smokers in the DCC set. However, this classifier did not show improved sensitivity in identifying never-smokers across the seven data sets compared to the original DCC-classifier (data not shown), in line with findings by Landi et al. [13] that lung cancer gene expression is to a large extent similar in current and former smokers.

**Table 3:** Identification of never-smokers based on supervised analysis of never-smokers versus smokers in seven AC data sets

	**DCC^A^**	**GSE10072^A^**	**GSE11969^A^**	**GSE12667^A^**	**GSE32863^A^**	**Beer^A^**	Illumina^A^*
DCC centroids	88%**	88%	93%	100%	86%	89%	80%
GSE10072 centroids	90%	100%**	87%	100%	76%	100%	80%
GSE11969 centroids	78%	88%	89%**	100%	59%	89%	80%
GSE12667 centroids	76%	88%	82%	100%**	66%	56%	60%
GSE32863 centroids	78%	81%	93%	88%	97%**	100%	90%
Beer centroids	84%	94%	87%	100%	76%	100%**	70%
Illumina centroids *	76%	75%	89%	88%	76%	78%	100%**

^A^ Sensitivity in identifying true never-smokers of a supervised classifier (rows) when applied to a specific data set (columns).

* The original cohort of 39 AC.

** Classifier applied to its own training data.

To investigate whether classification sensitivity and specificity could be improved we applied a series of more stringent classification thresholds for the DCC-derived classifier specifically (see Methods). Notably, increased classification stringency improved sensitivity only slightly, specificity less, while introducing a large number of unclassified samples across the seven tested data sets for this classifier (in Additional file 3: Figure S1). Notably, in the DCC, GSE10072, GSE12667, GSE11969, Beer et al., GSE32863, and original Illumina cohort 87%, 92%, 71%, 82%, 80%, 78% and 95%, respectively, of samples classified as never-smokers by the DCC classifier were also classified as AC1. Moreover, analysis of pack-year data from Beer et al., GSE32863, and GSE11969 revealed no significant difference between smokers classified differently by the DCC-classifier (p > 0.05 all comparisons, Student’s *t*-test). Taken together, these comparisons indicate that the unsupervised and supervised approaches both identify a core set of samples as “potential never-smokers” that comprises both true never-smokers and smokers, with the latter including both current and former smokers.

### Functional analysis of gene signatures from unsupervised and supervised analysis

Functional analyses of the AC1/AC2 and DCC-derived gene signatures were performed using LitVAn [24] and IPA. For the AC1/AC2 signature LitVAn analysis revealed that genes with lower expression in AC1 showed enrichment for only a few gene ontology terms, e.g., fibrinogen. In contrast, LitVAn, and IPA both identified a strong association of genes overexpressed in AC1 with different immunological functions (in Additional file 4: Table S3).

LitVAn analyses of the centroid classifiers from supervised analysis showed that terms associated with proliferation were the main functional associations of classifiers derived from analysis of the DCC (in Additional file 4: Table S3), GSE12667, and GSE10072 data sets. The strong influence of proliferation was further highlighted by the marked differences in CIN70 metagene expression between classification groups for the DCC classifier across investigated data sets (in Additional file 5: Figure S2). Notably, the AC1/AC2 classification showed a similar CIN70 expression pattern as the DCC classifier across the majority of data sets, with lower expression in the AC1 group harboring the true never-smokers, despite differences in functional associations (in Additional file 5: Figure S2). This similarity in CIN70 expression is likely explained by the previously described high overlap between the two classifiers. Moreover, in the GSE11969 data set, representing the only external data set with *EGFR*, *KRAS*, and *TP53* mutation data, both the AC1/AC2 signature and the DCC derived classifier were strongly associated with *EGFR* mutations (p = 0.002 and 0.001 respectively, Fisher’s exact test), but not with *KRAS* or *TP53* mutations. In further support of the latter finding, the AC1/AC2 signature and the DCC-classifier were also not associated with p53 status or *KRAS* mutations in the Beer et al. data set.

### Association of tumor derived gene signatures with smoking history in normal airway epithelial samples and adjacent lung tissue

To further investigate results from unsupervised and supervised analysis of tumor cases we applied the AC1/AC2 gene signature and the DCC classifier to four data sets comprising 360 gene expression profiles of histologically normal bronchial airway epithelial specimens. Convincingly, for both the AC1/AC2 signature and the DCC classifier similar results were obtained as for classification of AC data sets, i.e. high sensitivity in identifying never-smokers however with a notable fraction of smoker cases classified as “potential never-smokers” (Table 4). Stratification of smokers into former or current smokers showed that 55–61% of former smokers and 23–54% of current smokers were classified as AC1 across the different data sets, while corresponding numbers for the DCC-classifier were 45–75% and 21–40% (Table 4). Analysis of pack-year data from smokers in GSE19027, GSE7895, GSE19667 and GSE11952 revealed no significant difference between AC1 smokers compared to AC2 smokers, or for smokers classified differently by the DCC-classifier (p > 0.05 all comparisons, Student’s *t*-test, in Additional file 6: Figure S3). For former smokers in GSE7895 with available data on time since smoking cessation there was no difference between AC1 and AC2 classified cases, or between DCC-classification (p = 0.46 and p = 0.14, respectively, Wilcoxon’s test). Moreover, there was no difference between classifications (AC1/AC2, DCC-classifier) regarding whether smokers in GSE19027 developed cancer or not (p = 0.38, AC1/AC2 and p = 0.40 DCC-classifier, Fisher’s exact test). Overlap between the two classifications were lower in the normal airway data sets compared to AC data sets, as 49%, 64%, 55%, and 61% of cases classified as never-smokers by the DCC derived signature were also classified as AC1 in GSE7895, GSE19027, GSE19667, and GSE11952 respectively. The higher discrepancy between classifications was also evident in the expression of the CIN70 metagene in the four data sets (in Additional file 7: Figure S4).

**Table 4:** Association of the AC1/AC2 and DCC signature with smoking history in normal airway epithelial samples, and adjacent lung tissue

**Data set**	Number NS/S/CS/FS*	**Number NS classified as AC1**	**% S/CS/FS classified as AC1**	P-value AC1/AC2 classification**	Number correctly identified NS by DCC signature***	**% S/CS/FS classified as never-smokers by DCC signature**	P-value DCC classification**
GSE19027	8/44/24/20	7 (88%)	55/54/55	0.12	8 (100%)	45/21/75	0.005
GSE7895	21/83/52/31	16 (76%)	37/23/61	0.003	16 (76%)	42/40/45	0.007
GSE19667	48/73/NA/NA	38 (79%)	33/NA/NA	8 × 10^−7^	31 (65%)	33/NA/NA	0.0008
GSE11952	38/45/45/0	29 (76%)	29/29/0	2 × 10^−5^	21 (55%)	38/38/0	0.13
GSE32863^A^	30/28/28/0	23 (77%)	39/39/0	0.007	24 (80%)	39/39/0	0.003
GSE10072^A^	15/34/16/18	12 (80%)	44/38/50	0.03	13 (87%)	44/38/50	0.01

* NS: never-smoker, S: smoker, CS: current smoker, FS: former smoker.

** Fisher’s exact P-value calculated from a 2×2 contingency table summarizing number of never-smokers and smokers for each classification group.

*** NS classified as never-smoker by the classifier.

^A^ Adjacent normal lung tissue data set.

Moreover, we also investigated the AC1/AC2 and DCC classifiers in normal adjacent lung tissue (n = 107) included in two of the AC data sets (GSE32863 and GSE10072). Notably, results for the AC1/AC2 classification and the DCC classifier were in line with the four normal airway epithelial data sets (Table 4). Again, analysis of pack-years in GSE32863 revealed no difference between AC1-smokers and AC2-smokers, or for the DCC-classifier (p = 0.35 and p = 0.08, respectively, Student’s *t*-test, in Additional file 6: Figure S3). Moreover, overlap between AC1/AC2 and DCC classifications were similar to the airway data sets as 61% and 69% of cases classified as never-smokers by the DCC derived signature were also classified as AC1 in GSE10072 and GSE32863 respectively.

## Discussion

The genetic basis for initiation and development of lung carcinoma has a clinical impact through targeted therapeutics, diagnostic tools, prognostics, and predictive markers. Gene expression and genomic profiling have been used extensively in lung cancer to dissect the diversity of the disease and to derive prognostic gene signatures [5,6,8,10,25,26]. Furthermore, such high throughput studies have also been performed to identify gene signatures associated with cigarette smoking in both tumor and bronchial epithelial tissue [13,15,16]. Indeed, lung cancer in never-smokers is among the top ten causes of cancer mortality in the world and successful genome-wide characterization of lung cancer stratified by patients’ smoking history may have large future implications for evaluation of lung cancer risk in the absence of smoking. However, although lung cancer in never-smokers has been suggested to represent a different disease entity compared to cancers arising in smokers [2,3], numerous reports of gene expression derived AC subtypes have reported consistent lack of a never-smokers’ or a never-smoker predominant AC subtype [5-10].

In the current study we aimed to delineate transcriptional differences between AC arising in smokers and never-smokers in seven AC data sets by both unsupervised and supervised gene expression analysis. Notably, these data sets were analyzed by different microarray platforms and represent patient materials of different stage, differentiation, ethnicity, age, and sex. Our initial unsupervised analysis of a small, but well characterized AC cohort (n = 39) broadly divided cases into two main subgroups termed AC1 and AC2 (Figure 1). Intriguingly, AC1 harbored all never-smokers together with more than half of AC smoker cases, including both current and former smokers. We next validated the association of the AC1 group with never-smoking patient status through a derived gene expression signature in six larger external AC data sets (Table 2) and, notably, across all validation sets, confirmed the existence of an AC1 profile displaying roughly similar proportions of smokers (current/former) and never-smokers as in the original cohort (Table 2). Importantly, although the gene signature for the AC1 and AC2 subgroups was derived from initial analysis of a small cohort comprising only nine never-smokers, it was successfully validated across much larger AC data sets, e.g., the DCC (n = 349), profiled by different microarray platforms and comprising in total 687 AC tumor cases. Moreover, characteristics of the AC1 and AC2 groups appear consistent with findings from several studies demonstrating differences between smokers and never-smokers with AC. This includes association with female sex in two of the external AC data sets (DCC and GSE10072, data not shown), successful validation in patient cohorts of different ethnicity, higher proliferation in smoking compared to never-smoking cases within AC1 [13], and association of AC1 with increased EGFR activity (GSE11969 and our original data). Moreover, in line with subtypes reported by Takeuchi et al. [9] AC1 cases in GSE11969 were more often classified as terminal respiratory unit (TRU) -type AC (p = 0.03, Fisher’s exact test) proposed to represent a subgroup of AC originating from the peripheral airway epithelium under less influence of smoking and retaining certain progenitor characteristics [9].

Motivated by the high sensitivity, but lower specificity, in identification of never-smokers by the AC1/AC2 gene signature generated from unsupervised analysis, we also performed supervised analysis between never-smokers and smokers in seven AC data sets (n = 726). For each data set, we identified differentially expressed genes that we used to generate a centroid classifier, which we subsequently used to classify all data sets. Interestingly, the centroid classifiers with the best sensitivity in identifying never-smokers across the seven AC data sets (i.e. classifiers from the DCC and GSE10072 data sets) showed similar performance as the corresponding AC1/AC2 classification (Tables 2 and 3). In line with our original findings from the unsupervised clustering, all centroid classifiers derived from supervised analysis grouped a notable fraction of smokers as potential “never-smokers”, including both current and former smokers (in Additional file 2: Table S2). Moreover, there was a strong overlap of samples classified as never-smokers by the DCC-derived classifier and by the AC1/AC2 classification across all analyzed tumor data sets. This overlap indicates that the two approaches identify a core set of samples as potential never-smokers that comprise both true never-smokers and smokers. Thus, despite differences in the type of analysis, in size of original data sets generating the classifiers, and in apparent functional associations of the two signatures, a consistency regarding classification of AC stratified by smoking history could indeed be demonstrated by the two approaches herein. These results could indicate the existence of a potential molecular subtype of AC with a presumed non-smoking-associated etiology. Landi et al. recently proposed a gene expression signature characteristic of smoking, heavily weighted on cell cycle genes, that separated both smokers from non-smokers in lung tumors and early stage tumor tissue from non-tumor tissue [13]. Interestingly, the DCC-classifier showed considerable overlap with results from Landi et al. [13]. Specifically, seven out of the 20 up-regulated genes reported by Landi et al. were present in the DCC-derived classifier, including nearly all of the genes involved in regulation of mitotic spindle formation highlighted by Landi et al. as an important pathway deregulated between AC arising in smokers and never-smokers. In contrast, none of 20 up-regulated genes reported by Landi et al. were present in the AC1/AC2 signature. Moreover, an average metagene expression value of the 20 up-regulated genes in the Landi signature showed a Pearson correlation of 0.99 with corresponding CIN70 expression values for cases in the original Illumina cohort (data not shown). This correlation suggests that the coherent pattern of DCC classification with CIN70 expression (DCC classified smokers high CIN70, DCC classified never-smokers low CIN70 expression) resembles findings by Landi et al. [13]. However, despite that classification by the supervised DCC classifier to a large extent appear coherent with expression of proliferation associated genes (in Additional file 5: Figure S2), specificity in identifying never-smokers remained low to medium even when markedly increasing the classification threshold in the seven AC data sets, (in Additional file 3: Figure S1).

Interestingly, when the AC1/AC2 and DCC classifiers were applied to four data sets comprising histologically normal airway epithelial tissue (n = 360 cases), and two data sets with normal adjacent lung tissue (n = 107) sensitivity in detecting never-smokers were high for both tumor-derived classifiers. However, similar to the tumor analysis never-smokers could not be singled out as unique group (Table 4). Cigarette smoke exposure has been demonstrated to create a “field of injury” in airway epithelial cells [27], and genes involved in regulation of oxidant stress, xenobiotic metabolism, and oncogenesis have been reported to be induced by smoking, while genes involved in tumor suppression and inflammation pathways have been reported to be down regulated [28]. The latter, in combination with findings by Landi et al. [13] that current smoking altered expression of immune response associated genes in non-tumor tissue, appears consistent with the functional association of the AC1/AC2 signature (in Additional file 4: Table S3). Moreover, expression of several genes in the AC1/AC2 signature appear consistent with reports about gene expression changes in relation to smoking in airway epithelial cells. E.g., two (*CX3CL1* and *PLA2G10*) of the 13 genes reported to be irreversibly altered by cigarette smoke by Spira et al. [28] are present in the AC1/AC2 gene signature. *CX3CL1,* a well-known chemokine, was found to be irreversibly downregulated in smokers [28], consistent with its lower expression in AC2 cases, while *PLA2G10* was found irreversibly upregulated in smokers [28], in line with its elevated expression in AC2 cases. Moreover, *MUC5ACGPX2UCHL1*, and *CABYR* have all been associated with increased expression in smokers compared to never-smokers, in line with their higher expression in the AC2 centroid compared to the AC1 centroid (in Additional file 1: Table S1) [28-30]. In addition to genes associated with smoking the AC1/AC2 classifier included several genes implicated in lung cancer tumorigenesis, such as *KITID1MMP7MYCNXRN2*, and *CYP24A1*, as well as type II pneumocyte marker genes such as *NKX2-1* (*TTF1*/*TITF1*), *LAMP3* (*CD208*), and surfactant proteins *SFTPB* and *SFTPC* (in Additional file 1: Table S1). Type II pneumocytes have an intriguing role in lung disease, as anomalies in pulmonary surfactant protein levels have been associated with certain respiratory diseases frequently observed in smokers [31]. Moreover, type II pneumocytes in the alveoli of the lung have been associated with progenitor-like characteristics due to their ability to regenerate the alveolar epithelium after injury and also play an important role in the innate immune response of the lung through secretion of surfactant proteins and different proinflammatory mediators [32,33]. Notably, the DCC-derived classifier also included, besides genes associated with proliferation, genes reported to be affected by smoking in airway epithelial cells, such as *CX3CL1GPX2UCHL1HLF*[28], *CYP1B1*[28], and *S100A8*[34], with expression consistent with previous reports. In summary, the findings of a considerable number of reported smoking induced genes with consistent expression in the tumor-derived gene signatures suggest that these signatures are in fact related to patient smoking history. However, whether the relationship is due to expression differences in the tumor cells or the surrounding stromal tissue remains to be determined, as delineation of the expression from non-microdissected heterogeneous tissue is highly problematic.

Taken together, results from the current study in combination with previous reports on different AC subtypes [6,7,9,10] indicate that never-smokers can not be completely separated from smokers based on transcriptional differences, and consequently, that AC arising in never-smokers do not appear to represent a distinct entity based on transcriptional patterns. Instead, this may suggest a shared biology between AC arising in never-smokers and in a subgroup of smokers, the latter thus perhaps representing tumors that arised in smokers “by chance”, i.e., possibly independent, or less dependent, of a positive smoking history, which warrants further investigation.

## Conclusions

In the current study we have sought to identify transcriptional patterns specific for never-smokers with AC compared to tumors arising in smokers. Both unsupervised and supervised gene expression analysis identified simple classifiers (harboring both smoking induced genes and genes implicated in lung tumorigenesis) with high sensitivity in identifying never-smokers across multiple AC and normal tissue data sets. Furthermore, and consistent between original and validation data sets, a subset of tumors arising in smokers (both current and ex) was classified together with tumors arising in never-smokers, thus together forming a subgroup of AC with shared transcriptional patterns and, as discussed above, also other strong similarities. Taken together, these analyses provide further insight into the heterogeneous transcriptional patterns occurring in AC stratified by smoking history.

## Competing interests

The authors declare that they have no competing interests.

## Author’s contributions

MP conceived of the study. JS, GJ, and MP wrote the manuscript. JS and GJ performed data analysis. MJ and AK performed array experiments. MJ, AS, SI, and MP performed IHC and mutational analysis. SBE, PJ, MS, and HP included the patients and contributed with tumor material. LJ initiated consecutive bio banking of fresh frozen lung cancer tissue, did most of the tissue sampling and performed the histopathological examinations. All authors approved the final manuscript.

## Pre-publication history

The pre-publication history for this paper can be accessed here:

http://www.biomedcentral.com/1755-8794/5/22/prepub

## Supplementary Material

Additional file 1 **Table S1. **AC1/AC2 and supervised gene expression centroids. An Excel file, Table S1, containing gene expression centroids for the AC1/AC2 and seven gene signatures derived from supervised analysis. Click here for file

Additional file 2 **Table S2. **Fraction of smokers, subdivided also into current and former status, classified as never-smokers by classifiers derived from supervised analysis of seven AC data sets. An Excel file, Table S2, describing the fraction of true smokers overall, current smokers, and former smokers classified as never-smokers by classifiers derived from supervised analysis of seven AC data sets. Click here for file

Additional file 3 **Figure S1. **Sensitivity and specificity of the DCC derived classifier for identification of never-smokers across different correlation classification cut-offs. A pdf file, Figure S1, showing the sensitivity and specificity of the DCC derived classifier for identification of never-smokers across different correlation classification cut-offs in seven data sets. Sensitivity and specificity for different Pearson correlation classification cut-offs are shown in the left subpanels, while the corresponding number of DCC-classified never-smokers and smokers are shown in the right panel for respective data set. A) DCC-classifier applied to the DCC data set. B) DCC-classifier applied to GSE10072. C) DCC-classifier applied to GSE12667. D) DCC-classifier applied to GSE11969. E) DCC-classifier applied to Beer et al. F) DCC-classifier applied to GSE32863. G) DCC-classifier applied to the original 39 adenocarcinomas. Click here for file

Additional file 4 **Table S3. **Functional analysis of AC1/AC2 gene signature derived from unsupervised analysis, and the classifier derived from supervised analysis of the DCC data set using LitVAn and IPA. An Excel file, Table S3, showing results from Functional analysis of AC1/AC2 gene signature derived from unsupervised analysis, and the classifier derived from supervised analysis of the DCC data set using LitVAn and IPA. Click here for file

Additional file 5 **Figure S2 **Expression of the CIN70 metagene across seven AC data sets classified by both unsupervised and supervised analysis. A pdf file, Figure S2, showing the log2ratio expression of the CIN70 metagene across seven AC data sets classified by both unsupervised and supervised analysis. CIN70 metagene expression displayed as box plots for true never-smokers and smokers (white), true current, former and never-smokers (gray), AC1 and AC2 classified samples (blue), and DCC centroid classified samples (red) in A) the DCC data set, B) GSE10072, C) GSE11969, D) GSE12667, E) Beer et al., F) GSE32863, and G) the original Illumina cohort of 39 AC. P-values were calculated using Wilcoxon’s test (two groups) or Kruskal-Wallis test (three groups). Click here for file

Additional file 6 **Figure S3. **Pack-year analysis of five data sets comprising normal airway epithelial cells or normal adjacent lung tissue classified by both unsupervised and supervised analysis. A pdf file, Figure S3, showing pack-year distribution for classification of five data sets using classifiers from unsupervised and supervised analyses. Pack-years for AC1/AC2 classification (**A**) or DCC-classification (**B**) for GSE7895, GSE11952, GSE19027, GSE19667 and GSE32863 respectively. P-values calculated using either Student’s *t*-test or Wilcoxon’s test. Click here for file

Additional file 7 **Figure S4. **Expression of the CIN70 metagene across six data sets comprising normal airway epithelial cells or normal adjacent lung tissue classified by both unsupervised and supervised analysis. A pdf file, Figure S4, showing the expression of the CIN70 metagene across six data sets comprising normal airway epithelial cells or normal adjacent lung tissue classified by both unsupervised and supervised analysis. CIN70 metagene log2ratio expression are displayed as box plots for true never-smokers and smokers (white), true current, former and never-smokers (gray), AC1 and AC2 classified samples (blue), and DCC centroid classified samples (red) in A) GSE7895, B) GSE19027, C) GSE19667, D) GSE11952, E) normal samples in GSE10072, and F) normal samples in GSE32863. P-values were calculated using Wilcoxon’s test (two groups) or Kruskal-Wallis test (three groups). Click here for file
