# Supplementary figures and images for: Relation between smoking history and gene expression profiles in lung adenocarcinomas
Source: BMC Med Genomics. 2012 Jun 7;5:22. doi: 10.1186/1755-8794-5-22 (PMC3447685; doi:10.1186/1755-8794-5-22)

**A**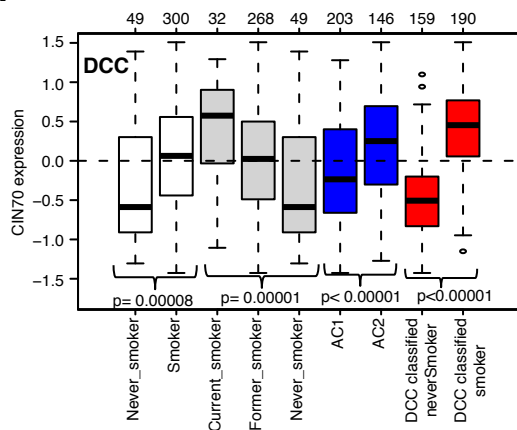**B**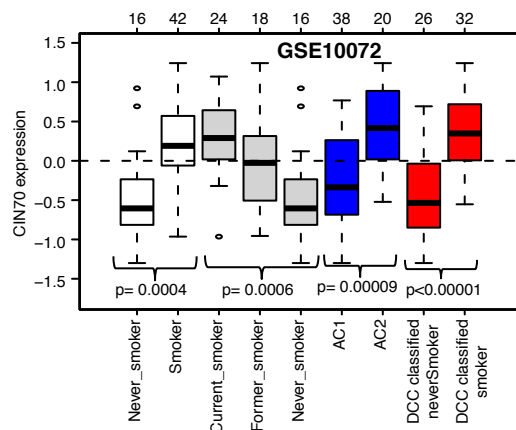**C**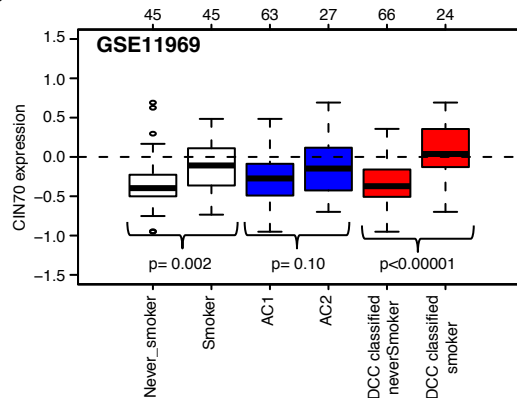**D**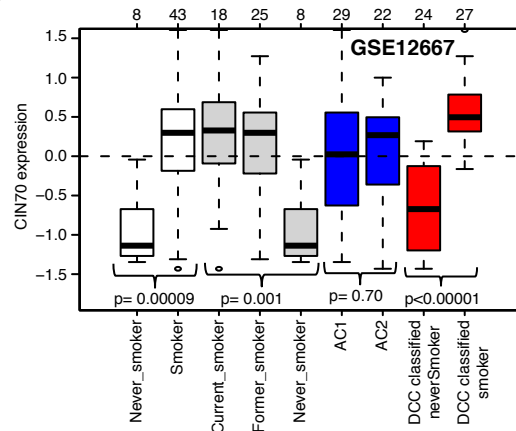**E**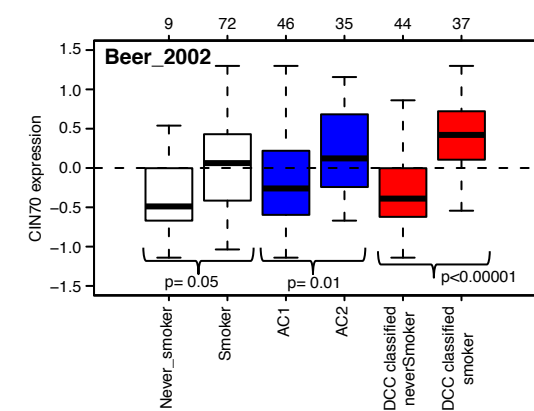**F**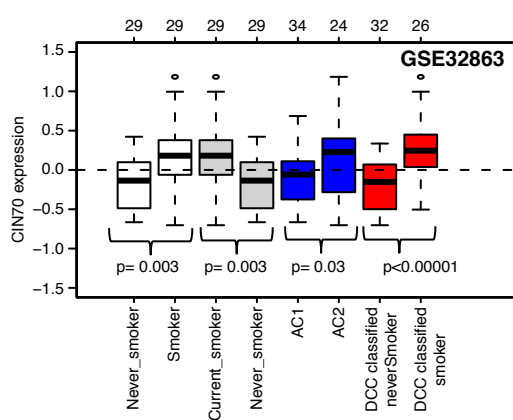**G**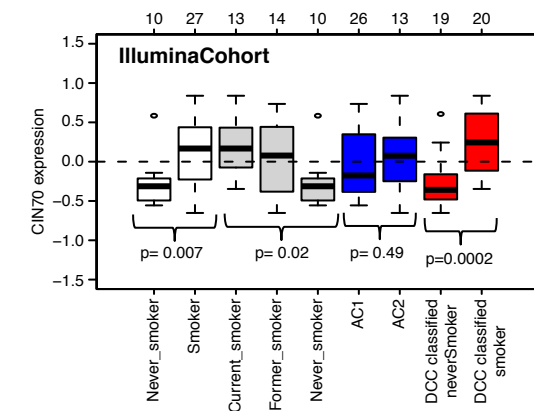

Supplement: Additional file 5 — Figure S2 Expression of the CIN70 metagene across seven AC data sets classified by both unsupervised and supervised analysis. A pdf file, Figure S2, showing the log2ratio expression of the CIN70 metagene across seven AC data sets classified by both unsupervised and supervised analysis. CIN70 metagene expression displayed as box plots for true never-smokers and smokers (white), true current, former and never-smokers (gray), AC1 and AC2 classified samples (blue), and DCC centroid classified samples (red) in A) the DCC data set, B) GSE10072, C) GSE11969, D) GSE12667, E) Beer et al., F) GSE32863, and G) the original Illumina cohort of 39 AC. P-values were calculated using Wilcoxon’s test (two groups) or Kruskal-Wallis test (three groups). [file 1755-8794-5-22-S5.pdf]

**A**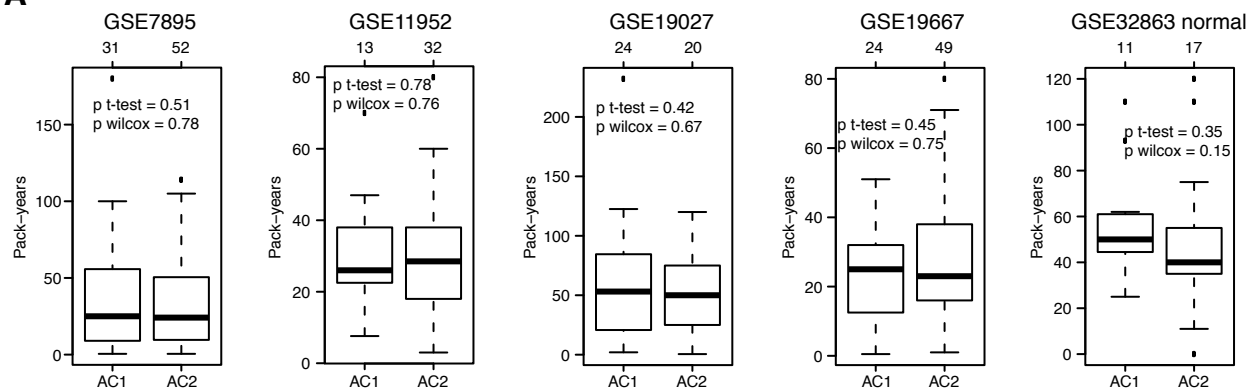**B**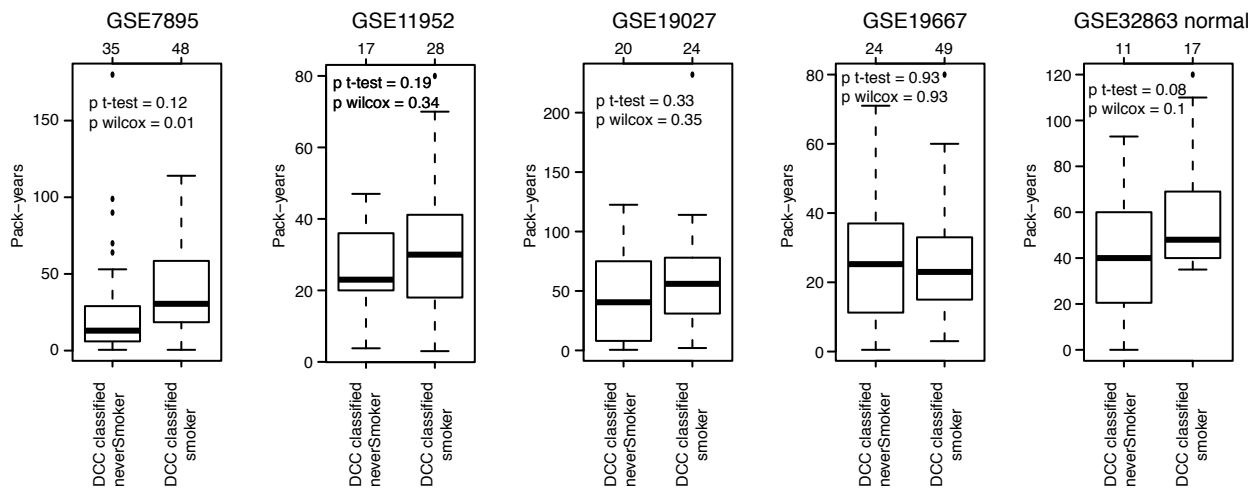

Supplement: Additional file 6 — Figure S3. Pack-year analysis of five data sets comprising normal airway epithelial cells or normal adjacent lung tissue classified by both unsupervised and supervised analysis. A pdf file, Figure S3, showing pack-year distribution for classification of five data sets using classifiers from unsupervised and supervised analyses. Pack-years for AC1/AC2 classification (A) or DCC-classification (B) for GSE7895, GSE11952, GSE19027, GSE19667 and GSE32863 respectively. P-values calculated using either Student’s t-test or Wilcoxon’s test. [file 1755-8794-5-22-S6.pdf]

**A**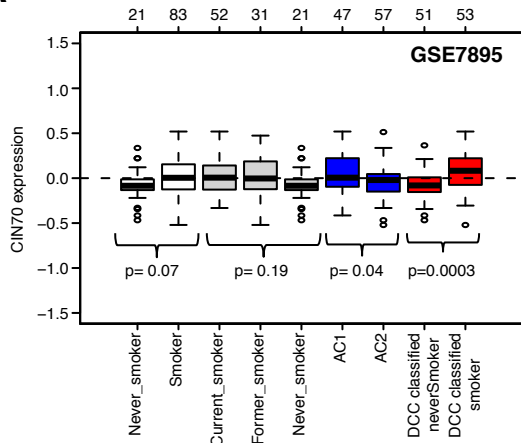**B**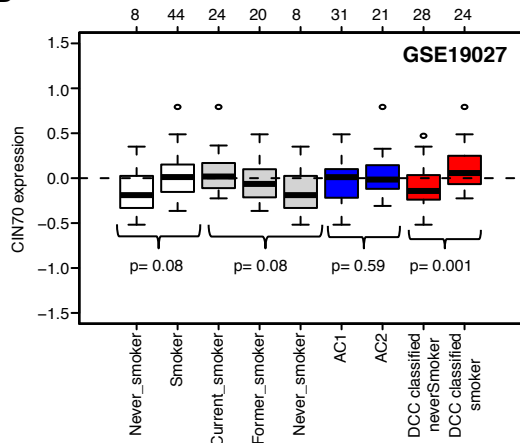**C**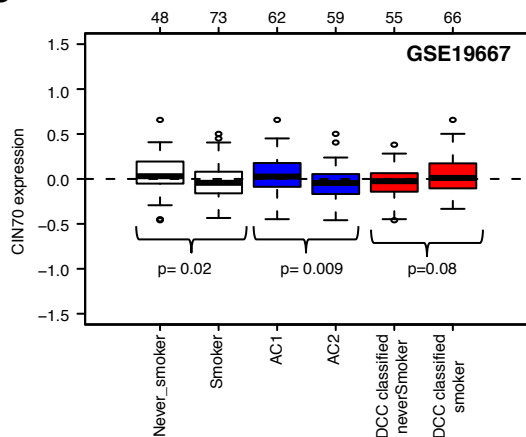**D**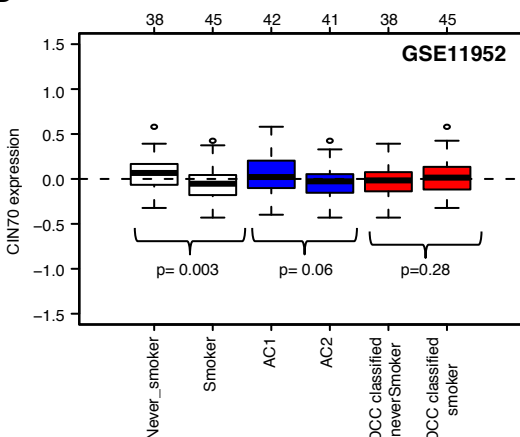**E**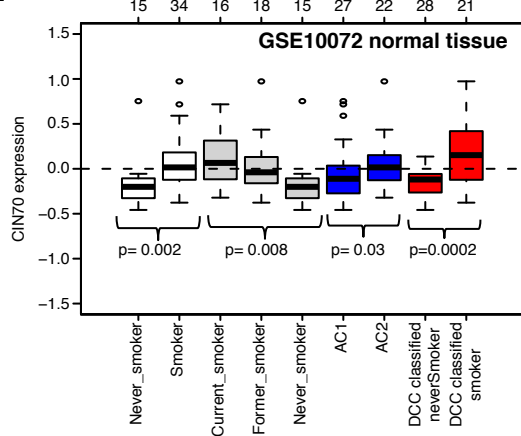**F**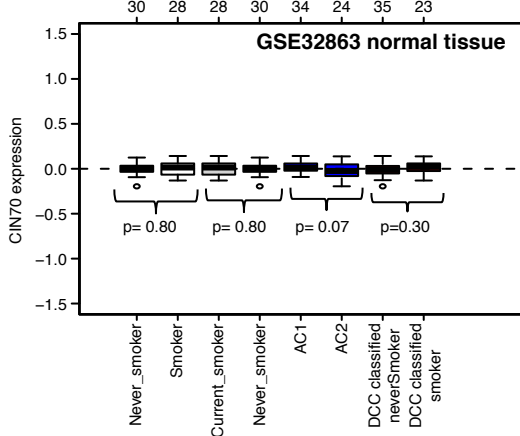

Supplement: Additional file 7 — Figure S4. Expression of the CIN70 metagene across six data sets comprising normal airway epithelial cells or normal adjacent lung tissue classified by both unsupervised and supervised analysis. A pdf file, Figure S4, showing the expression of the CIN70 metagene across six data sets comprising normal airway epithelial cells or normal adjacent lung tissue classified by both unsupervised and supervised analysis. CIN70 metagene log2ratio expression are displayed as box plots for true never-smokers and smokers (white), true current, former and never-smokers (gray), AC1 and AC2 classified samples (blue), and DCC centroid classified samples (red) in A) GSE7895, B) GSE19027, C) GSE19667, D) GSE11952, E) normal samples in GSE10072, and F) normal samples in GSE32863. P-values were calculated using Wilcoxon’s test (two groups) or Kruskal-Wallis test (three groups). [file 1755-8794-5-22-S7.pdf]
