# Supplementary material for: Relation between smoking history and gene expression profiles in lung adenocarcinomas
Source: BMC Med Genomics. 2012 Jun 7;5:22. doi: 10.1186/1755-8794-5-22 (PMC3447685; doi:10.1186/1755-8794-5-22)
Supplement: Additional file 3 — Figure S1. Sensitivity and specificity of the DCC derived classifier for identification of never-smokers across different correlation classification cut-offs. A pdf file, Figure S1, showing the sensitivity and specificity of the DCC derived classifier for identification of never-smokers across different correlation classification cut-offs in seven data sets. Sensitivity and specificity for different Pearson correlation classification cut-offs are shown in the left subpanels, while the corresponding number of DCC-classified never-smokers and smokers are shown in the right panel for respective data set. A) DCC-classifier applied to the DCC data set. B) DCC-classifier applied to GSE10072. C) DCC-classifier applied to GSE12667. D) DCC-classifier applied to GSE11969. E) DCC-classifier applied to Beer et al. F) DCC-classifier applied to GSE32863. G) DCC-classifier applied to the original 39 adenocarcinomas. [file 1755-8794-5-22-S3.pdf]

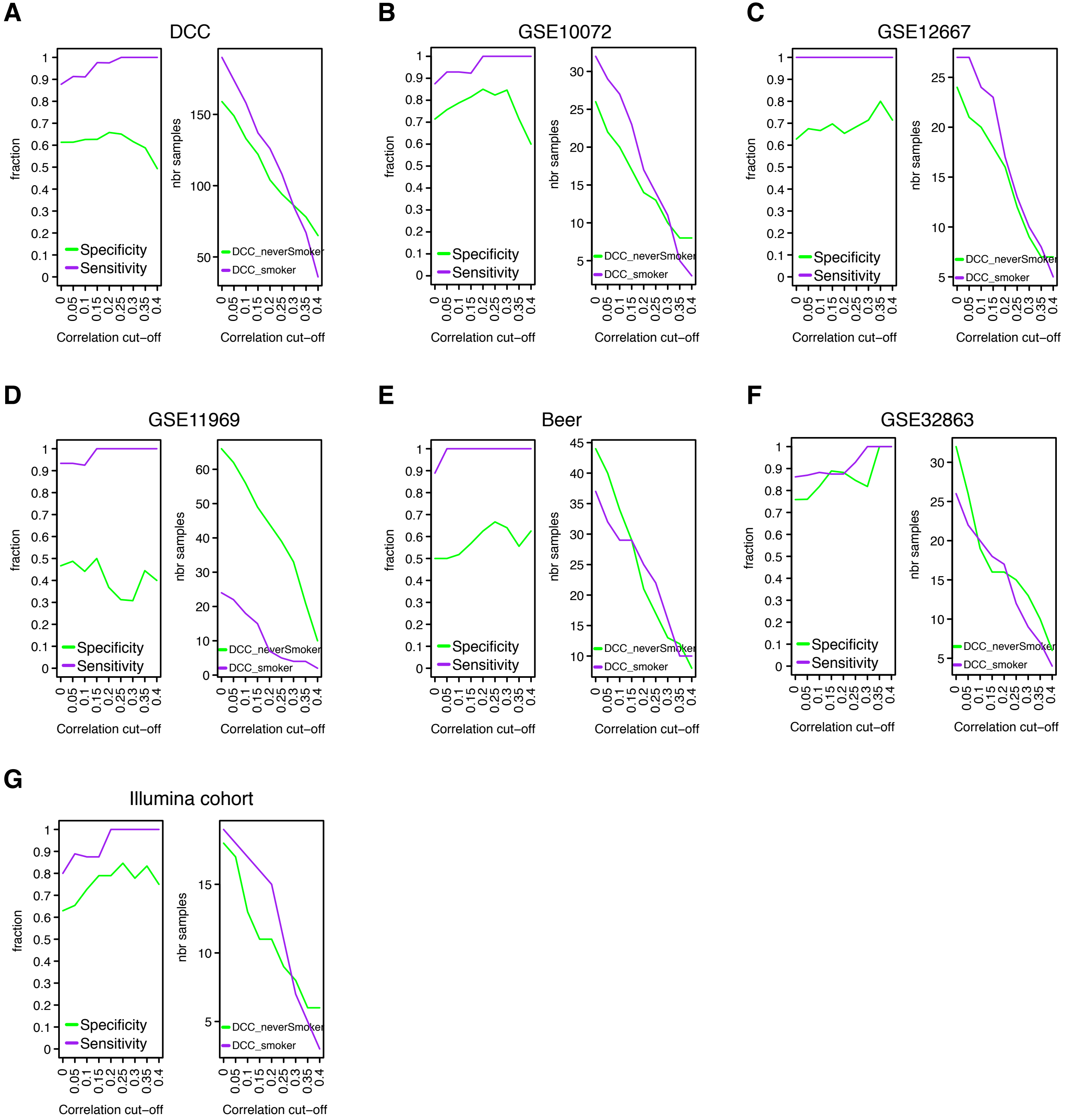

A
DCC
B
GSE10072
C
GSE12667
D
GSE11969
E
Beer
F
GSE32863
G
Illumina cohort
fraction
nbr samples
Specificity
Sensitivity
DCC_neverSmoker
DCC_smoker
Correlation cut-off
